# Correction: Exploring the physical, mental, and social dimensions of middle-aged adults for active and healthy aging: A cross-sectional study

**DOI:** 10.1371/journal.pone.0348375

**Published:** 2026-04-28

**Authors:** The PLOS One Staff

The Funding statement for this article is incorrect. The correct Funding statement is as follows: This work is funded by national funds through the Foundation for Science and Technology, under the project UID/06291/2025. Funding was received by all authors.

The publisher apologizes for the error.
