# Supplementary material for: Exploring the physical, mental, and social dimensions of middle-aged adults for active and healthy aging: A cross-sectional study
Source: PLoS One. 2025 Apr 2;20(4):e0320309. doi: 10.1371/journal.pone.0320309 (PMC11964271; doi:10.1371/journal.pone.0320309)
Supplement: Attachment — Submitted filename: responses to Reviewer 2.docx [file pone.0320309.s001.docx]

Dear Reviewer #2,

We would like to thank you for your valuable feedback and the insightful suggestions that you provided. Your comments were instrumental in improving the clarity and depth of the manuscript. Below, we address each of the points you raised and outline the revisions made in response.

a) Strengthening of the research with statistical data on ageing in Portugal: We appreciate your suggestion to incorporate statistics on ageing in the Portuguese population, particularly in the context of the Baixo Alentejo. In response, we have enhanced the introduction of the manuscript by including relevant demographic data on the region's population decline and ageing trends. This addition helps contextualise the importance of the study and the challenges faced by this region. The revised text is as follows:

*"The Alentejo region, which includes the Baixo Alentejo, could see a significant decline in population between 2000 and 2050, with the resident population falling by 46.1 per cent [1]. In particular, the young population is expected to fall by 70.4 per cent and the number of pre-school children by 72.9 per cent [1]. This makes it the Portuguese region with the highest ageing rate (189.2 per cent) [1–3]. Baixo Alentejo (NUTS III) is a predominantly rural region with the largest surface area in the country (8,544.6 km²) and the lowest population density (14.7 inhabitants/km²) [3–5]. Demographic challenges are exacerbated by long distances between towns, which can exceed 120 km, and a limited and inefficient public transport network [3,6]. Geographical dispersion and transport barriers make it difficult to provide effective health care, especially for the most dependent [2,6]. In this context, the ageing of the population in the Baixo Alentejo is a critical challenge, highlighting the urgent need for strategies to mitigate the socio-economic and public health impact of this phenomenon [2,3,7].”*

This inclusion provides the necessary demographic context and strengthens the manuscript by reinforcing the urgency of addressing the challenges posed by population ageing in the region.

b) Clarification of the methodology and participant selection: We have clarified the methodology regarding the participant selection process, as suggested. The revision aims to make the description of the participant recruitment process more transparent and specific. The revised text is as follows:

*"Each person attending the health centre for a consultation was invited by the nurse to participate in the study. The nurse explained the aims of the study, and if the person agreed, they were given the questionnaire to complete at that time, with sufficient time allowed."*

We believe this revision now clearly explains the participant selection process and how individuals were recruited for the study.

c) Interpretation of results, particularly with regard to higher disability scores among divorced and single individuals: Thank you for your valuable comment regarding the interpretation of the results, particularly the higher disability scores observed among divorced and single individuals. We have expanded the discussion to address this point in more detail. The updated text is as follows:

*"The results highlight the strong link between socio-economic factors and mental well-being, and underline the significant impact of gender and employment status. Women and the unemployed reported higher levels of depressive symptoms, while men reported higher levels of life satisfaction. Economic stability was found to be positively associated with both mental well-being and life satisfaction, highlighting the crucial role of financial security in shaping perceptions of social support. These findings point to the need for policy interventions that address socio-economic inequalities. In particular, policies aimed at promoting economic stability and strengthening social support systems are essential for improving health outcomes, especially for vulnerable populations."*

This revision provides a more detailed interpretation of the results, particularly in relation to the disability scores and the implications for policy development.

d) Strengthening the policy recommendations: We have taken your recommendation to reinforce the information for public policy based on the results of the study. The revised conclusion now includes a more direct focus on policy recommendations aimed at addressing the issues identified in the study. The updated text is as follows:

*"Although global studies on ageing are extensive, there is a lack of integrative research addressing the physical, mental, and social well-being (three dimensions simultaneously) of middle-aged adults in Portugal, particularly in the Baixo Alentejo, where demographic changes and socio-economic challenges require targeted attention [1–4]. Existing studies mainly focus on the elderly population and rarely explore the intersectoral dimensions of well-being among middle-aged adults in this under-researched region [2,3,5,6]. This gap limits the development of policies and interventions tailored to regional needs. Given that middle age represents a pivotal window for preventive action, addressing physical, mental, and social dimensions during this stage is essential for promoting active ageing strategies [14].”*

This addition clarifies the importance of focusing on middle-aged adults and the need for policies that specifically target this demographic in light of the challenges faced by the Baixo Alentejo region.

We truly appreciate your thoughtful suggestions, which have greatly improved the quality of the manuscript. Should you have any further comments or require additional clarifications, we are happy to address them.

Thank you once again for your time and constructive feedback.

Kind regards,

Eunice Santos
